# Hyperspectral reflectance and agro-physiological traits for field identification of salt-tolerant wheat genotypes using the genotype by yield*trait biplot technique

**DOI:** 10.3389/fpls.2023.1165113

**Published:** 2023-08-02

**Authors:** Ahmed M. S. Elfanah, Mohamed A. Darwish, Adel I. Selim, Omnya M. A. Elmoselhy, Abdelraouf M. Ali, Maher A. El-Maghraby, Magdi T. Abdelhamid

**Affiliations:** ^1^ Wheat Research Department, Field Crops Research Institute, Agricultural Research Center, Giza, Egypt; ^2^ Food Crops Research Institute, Yunnan Academy of Agricultural Sciences, Kunming, China; ^3^ National Authority for Remote Sensing and Space Sciences (NARSS), Cairo, Egypt; ^4^ Department of Environmental Management, Institute of Environmental Engineering, People’s Friendship University of Russia (RUDN University), Moscow, Russia; ^5^ Botany Department, National Research Centre, Cairo, Egypt; ^6^ Department of Soil and Crop Sciences, Texas A&M University, College Station, TX, United States

**Keywords:** salinity tolerance indices, bread wheat, GGE biplots, GYT biplots, physiological traits, hyperspectral reflectance indices

## Abstract

**Introduction:**

Salinity is the abiotic obstacle that diminishes food production globally. Salinization causes by natural conditions, such as climate change, or human activities, e.g., irrigation and derange misuse. To cope with the salinity problem, improve the crop environment or utilize crop/wheat breeding (by phenotyping), specifically in spread field conditions. For example, about 33 % of the cropping area in Egypt is affected by salinity.

**Methods:**

Therefore, this study evaluated forty bread wheat genotypes under contrasting salinity field conditions across seasons 2019/20 and 2020/21 at Sakha research station in the north of Egypt. To identify the tolerance genotypes, performing physiological parameters, e.g., Fv/Fm, CCI, Na+, and K+, spectral reflectance indices (SRIs), such as NDVI, MCARI, and SR, and estimated salinity tolerance indices based on grain yield in non-saline soil and saline soil sites over the tested years. These traits (parameters) and grain yield are simultaneously performed for generating GYT biplots.

**Results:**

The results presented significant differences (P≤0.01) among the environments, genotypes, and their interaction for grain yield (GY) evaluated in the four environments. And the first season for traits, grain yield (GY), plant height (PH), harvest index (HI), chlorophyll content index (CCI), chlorophyll fluorescence parameter Fv/Fm, normalized difference vegetation index (NDVI) in contrasting salinity environments. Additionally, significant differences were detected among environments, genotypes, and their interaction for grain yield along with spectral reflectance indices (SRIs), e.g., Blue/Green index (BIG2), curvature index (CI), normalized difference vegetation index (NDVI), Modified simple ratio (MSR). Relying on the genotype plus genotype by environment (GGE) approach, genotypes 34 and 1 are the best for salinity sites. Genotypes 1 and 29 are the best from the genotype by stress tolerance indices (GSTI) biplot and genotype 34. Genotype 1 is the best from the genotype by yield*trait (GYT) method with spectral reflectance indices.

**Discussion:**

Therefore, we can identify genotype 1 as salinity tolerant based on the results of GSTI and GYT of SRIs and recommend involvement in the salinity breeding program in salt-affected soils. In conclusion, spectral reflectance indices were efficiently identifying genotypic variance.

## 1 Introduction

Soil salinity is an abiotic stressor and is considered one of the biggest obstacles to decreasing food production globally (Sardouie-Nasab et al., 2014; Tao et al., 2021; Quamruzzaman et al., 2022), impeding the breeding and releasing of cultivars (Bailey-Serres et al., 2019; Reynolds and Braun, 2022). It impacts more than 20%–40% of irrigated land (Ghonaim et al., 2021; Quamruzzaman et al., 2022). About 33% of the cropping area in Egypt is affected by salinity (Stavi et al., 2021; Morsy et al., 2022). Salinization is caused by either natural conditions (climate change) or human activities (anthropogenic, such as irrigation misuse) (Shabala et al., 2014; Stavi et al., 2021; Morsy et al., 2022). Reusing about 10 billion m^3^ of drainage water is considered a salinization source to increase soil salinity and reflects the limited water resources in Egypt (Mohamed, 2017). Hence, salinity is coped with through either crop environment management or crop improvement (breeding) (Rady et al., 2016; Alharbi et al., 2021; Morsy et al., 2022).

Wheat (*Triticum aestivum* L.) is a dominant cultivated cereal crop worldwide that has a role to play in food security. It contributes approximately 20% of human calories and protein (Reynolds and Braun, 2022). The total production is about 750 million tons annually. Nearly 9 million tons in Egypt is produced from 1.3 million hectares of wheat-cultivated areas (FAOSTAT, 2021). Breeding for stress tolerance acquires whether select tolerant genotype or maintenance of the environmental factors, such as reclamation of the soil, for example, adding gypsum to the soil (Stavi et al., 2021; Morsy et al., 2022), application of potassium fertilizer to enhance the salt-tolerance (Dawood et al., 2014), foliar spray of glutathione as an antioxidant with an organic biostimulant to improve the physiological and metabolic adaptation to salinity (ur Rehman et al., 2021) or improving wheat under biotic stress such as disease (Msundi et al., 2021), drought stress (Schneider et al., 1997; Abdelhakim et al., 2021), and salinity stress (Aycan et al., 2021; Ghonaim et al., 2021; Moustafa et al., 2021). In contrast, the selection (phenotyping) for stress tolerance purposes relies on the integration of multiple disciplines, not only agronomic traits (grain yield) but also physiological traits (Na^+^ and K^+^) (Oyiga et al., 2016; Tao et al., 2021) and hyperspectroscopic measurements (normalized difference vegetation index (NDVI) (Ali et al., 2018; Moghimi et al., 2018; Bruning et al., 2020). Salinity tolerance is a complex phenomenon controlled by several physiological traits and processes and genetic factors (Gizaw et al., 2018) and influenced by growth stages and open field conditions (Haq et al., 2010; Moghimi et al., 2018; Bruning et al., 2020). For breeding for wheat saline soil tolerance purposes, Sardouie-Nasab et al. (2014) reported field screening for 100 bread wheat genotypes under saline and non-saline conditions to identify tolerant genotypes utilizing salt tolerance indices (STIs) and principal component analysis (PCA). Moreover, several researchers (Hinojosa et al., 2019; Mohammadi et al., 2022) have screened a large set of genotypes and then selected appropriate genotypes for field stress evaluation.

Remote sensing technologies and spectral instruments create valuable spectral information in many wavelength bands throughout the electromagnetic spectrum, particularly visible, near-infrared, and shortwave, and provide spectral reflectance indices. These approaches are powerful tools for identifying chemical and physical plant structures and functions and are considered rapid, high-throughput, non-destructive, and accurate plant vegetation measurements (Reynolds et al., 2012; Sun et al., 2019; Bruning et al., 2020). Additionally, these techniques utilize spectral reflectance indices (SRIs) and phenotypic data (Gizaw et al., 2018). Plant phenotyping under field conditions and based on SRIs enables breeders to select improved vigorous and high-yielding genotypes (Hinojosa et al., 2019). Spectral reflectance indices assist in exploring various traits of plant vigor and performance and rely on visible (400–700 nm wavelength) and near-infrared spectra (>700 nm wavelengths), which are employed in plant phenotyping and screening, for example, NDVI (Santana et al., 2021), leaf greenness, and pigment abundance (Reynolds et al., 2012; Hinojosa et al., 2019). For instance, several vegetation indices were calculated based on canopy spectral properties, particularly for plants under stress, indicating agro-morphological traits indirectly (El-Hendawy et al., 2019), and the SRIs correlated with genotype Sakha 93 more than Sakha 61 for growth and grain yield. Sun et al. (2019) reported that applying canopy reflectance in winter wheat changed the canopy water content in different water regimes.

Physiological traits measured by instruments such as chlorophyll fluorescence are estimated as the Fv/Fm ratio (Hinojosa et al., 2019), and the chlorophyll content index (CCI) has been confirmed in plant phenotyping as a physiological trait measured by Opti-Sciences OS30p+ and Opti-Sciences CCM 200+ devices, respectively. For example, photosynthetic parameters decrease under saline conditions, which benefits the selection of salinity-tolerant genotypes (Munns and Gilliham, 2015; Tao et al., 2021). Additionally, Wu et al. (2015) pointed out the positive correlation between chlorophyll content and plant salt tolerance in barley and wheat (Tao et al., 2021). They reported that detecting chlorophyll fluorescence in the early growth stage is beneficial to preventing plant biomass loss under high-salinity treatment (El-Hendawy et al., 2019). In addition, researchers have reported that genotype selection based on genotype by trait (GT) biplots, including soil plant analysis development (SPAD) reading with agronomic traits, is an effective tool (Mohammadi, 2019; Kendal, 2020; Mohammadi and Amri, 2021). Salinity stress negatively influences chlorophyll fluorescence (Zarco-Tejada et al., 2003; Kalaji and Guo, 2008). On the other hand, physiological traits estimated in the laboratory that reflect a salinity-tolerant genotype include minimal Na^+^ concentration, higher K^+^ accumulation perfectly maintaining osmotic pressure in roots and shoots, and maximal photosystem (PSII) activities, resulting in producing higher biomass specifically under salinity stress (Oyiga et al., 2016; Morsy et al., 2022; Quamruzzaman et al., 2022).

STIs are used extensively in breeding programs (Sardouie-Nasab et al., 2014; Sabouri et al., 2022), and several applications compute them, such as *i*PASTIC (Pour-Aboughadareh et al., 2019). This online software generates several selection parameters, e.g., tolerance index (TOL) (Rosielle and Hamblin, 1981), mean productivity stress (MP) (Rosielle and Hamblin, 1981), STI (Fernandez, 1992), geometric mean productivity (GMP) (Fernandez, 1992), harmonic mean (HM) (Bidinger et al., 1987), stress susceptibility index (SSI) (Fischer and Maurer, 1978), yield index (YI), yield stability index (YSI) (Bouslama and Schapaugh, 1984), and relative stress index (Fischer and Wood, 1979). In addition, the combination of significant indices (CSI) (Sabouri et al., 2022) is proposed based on calculated means in contrasting conditions such as irrigated and non-irrigated environments. These parameters assist the breeders in selecting tolerant genotypes in cases studied to generate PCA, GT, and genotype by yield*trait (GYT) biplots (Sabouri et al., 2022; Bakhshi and Shahmoradi, 2023) for several crops.

The beneficial use of SRIs in wheat breeding programs is essential to recognize their relationship with agronomic traits (Santana et al., 2021). The GT and GYT biplot approaches (Yan et al., 2007; Yan and Frégeau-Reid, 2018; Darwish et al., 2023; Elfanah et al., 2023) allow breeders and statisticians to distinguish the correlation of traits and combinations, as well as the genotype rank and stability of these traits or GYT combinations. Furthermore, these biometrical techniques for identifying stress-tolerant genotypes rely on STIs. For example, GT, GYT, and genotype by yield*STI (GYSI) were employed to select barley drought-tolerant genotypes (Bakhshi and Shahmoradi, 2023), wheat breeds for stress tolerance (Mohammadi, 2019; Msundi et al., 2021; Zulfiqar et al., 2021), and other crops such as rice breeds for drought (Sabouri et al., 2022), barley (Kendal, 2020; Bakhshi and Shahmoradi, 2023), common bean genotypes (Sofi et al., 2022), and soybean (Kurbanov et al., 2023). Moreover, the GT model with SRIs in corn treated with nitrogen levels to identify trait relationships was used (Santana et al., 2021).

This study selected 40 elite genotypes based on evaluation of the previous season in multi-location trials. The selected genotypes were assessed in multisite and multi-season open fields under saline soil and non-saline conditions. The estimated characteristics were agronomic traits, physiological traits, STI computing parameters, and SRI. This study aims i) to evaluate and characterize 40 genotypes under saline and non-saline conditions to select salinity-tolerant genotypes and ii) to measure GYT using SRIs to assist wheat breeders in selecting genotypes positively correlated with grain yield to recommend the release of a new variety or to include it in a breeding program.

## 2 Materials and methods

### 2.1 Plant materials and experimental site

There were 40 genotypes selected from a local breeding program and exotic materials of CIMMYT yield trials, e.g., 39th ESWYT, 26th SAWYT, 26th HRWYT, and 8th SATYN (evaluated in multiple locations in Egypt in the 2017/18 season). These genotypes comprised 38 advanced breeding lines evaluated with two recently released cultivars, Misr 3 and Sakha 95, shown in **Table S1**.

Two separate field trials were carried out at the Sakha Agricultural Research Station, Kafr El-Sheikh, Egypt (latitude 31°5′N and longitude 30°56′E) in two successive cropping seasons, viz., 2019/2020 and 2020/2021. The Elhamrawy farm (part of the Sakha Agricultural Research Station) possesses saline soil with heavy clay (electrical conductivity (EC) ranging from 8.0 to 10.0 dS m^−1^), which could be classified as strongly saline soil. In addition, the Elnataf farm (another part of the Sakha Agricultural Research Station) is non-saline soil with heavy clay (EC ranging from 0.5 to 1.5 dS m^−1^), which could be classified as non-saline soil. Both experiments were irrigated with fresh water from an irrigation channel from the River Nile passing through the experimental area with pH 7.35 and EC of 0.41 dS m^−1^ using a surface irrigation system. Each experiment received five irrigations per season.

There were 40 genotypes planted in an alpha lattice experimental design with three replicates. Each plot consisted of six rows, 20 cm apart and 3.0 m long. Furthermore, the recommended phosphorus fertilizer dose was 35 kg P ha^−1^ before wheat sowing. At the same time, nitrogen fertilizer was added as 180 kg N ha^−1^ at each environment’s sowing and tillering stage. The sowing date was in the third week of November. These procedures are according to the Ministry of Agriculture, and Land Reclamation, Egypt. Some chemical characteristics of saline soil and non-saline sites in the 2019/2020 and 2020/2021 seasons are shown in **Table 1**.

**Table 1:** Some chemical characteristics of soil in non-saline soil and saline soil at the experimental sites before sowing in the 2019/2020 and 2020/2021 seasons.

1111	EC	pH	SAR	Soluble cations Meq/L				Soluble anions Meq/L			
	(dS/m)	(1:2.5)		Na^+^	Ca^++^	Mg^++^	K^+^	CO_3_^−^	HCO_3_^−^	Cl^−^	SO_4_^2−^
Non-saline/2020/2021	1.50	7.32	–	26.9	8.43	4.0	4.5	0.0	3.0	20.2	20.6
Saline/2019/2020	10.21	7.81	15.73	62.5	18.9	15.8	1.1	0.0	4.1	49.5	44.7
Saline/2019/2020	8.81	7.79	12.92	56.6	14.3	11.7	0.7	0.0	4.0	41.8	37.5
Saline/2020/2021	10.11	7.86	16.21	68.2	20.5	14.9	0.9	0.0	6.1	50.8	47.6
Saline/2020/2021	8.26	7.80	14.58	56.2	17.2	12.5	0.8	0.0	6.2	42.3	38.2

EC, the electrical conductivity of saturated extracts used (EC_e_); pH, soil acidity; SAR, sodium adsorption ratio.

### 2.2 The first season measured traits

The agronomic traits measured were days to maturity (DM), days to heading (DH), plant height (PH), number of spikes m^−1^ (SM), biomass or biological yield (BY), and grain yield (GY) kg h^−1^. Canopy temperature (CT) was obtained using a near-infrared temperature sensor (CEM DT 8835 infrared and K-type thermometer) at the completed flowering stage of each plot from 1:00 p.m. to 2:00 p.m. on a cloudless day. NDVI was measured by a field portable NDVI sensor (GreenSeeker® handheld crop sensor, Trimble Navigation Limited, Westminster, CO, USA). NDVI was measured between 11:30 a.m. and 2:00 p.m. The CCI was determined by a chlorophyll content meter (Opti-Sciences, Inc., CCM 200 plus) from the blade flag leaf (three readings per plot) at a completed flowering stage between 11 a.m. to 2 p.m. on a sunny day. Chlorophyll fluorescence (Fv/Fm) was estimated by a portable chlorophyll fluorometer (Opti-Sciences, OS30p_+_).

### 2.3 The second season measured traits

The agronomic traits measured were GY kg h^−1^. Additionally, the flame apparatus measured flag leaf concentrations of Na^+^ and K^+^. The leaf samples were collected from each plot at the end of the flowering stage. Furthermore, CT was measured as mentioned in the first season.

### 2.4 Spectroscopic measurements

#### 2.4.1 Spectral device

The hyperspectral reflectance of the wheat canopy was measured using a portable backpack ASD spectroradiometer (Analytical Spectral Devices Inc., Boulder, CO, USA), which captured the reflectance from 350 to 2,500 wavelength using an optical fiber probe. The measurement was within ±2 h of solar noon under cloudless conditions. Spectral reflectance indices and calculated equations are presented in **Table 2**.

**Table 2:** Spectral reflectance indices and calculation equations.

Vegetation index	Abbreviation	Formula	Reference
Normalized difference vegetation index	NDVI	(R_800_ − R_670_)/(R_800_ + R_670_)	(Rouse et al., 1974)
Modified chlorophyll absorption reflectance index	MCARI	((R_701_ − R_670_) − 0.2 (R_701_ − R_550)_)) × (R_701_/R_670_)	(Gamon and Surfus, 1999)
Leaf chlorophyll index	LCI	(R_850_) − (R_710_)/(R_850_) + (R_680_)	(Pu et al., 2008)
Curvature index	CI	R_675_ × R_690_/R^2^_683_	(Zarco-Tejada et al., 2003)
Triangular vegetation index	TVI	0.5(120 (R_750_ − R_550_) − 200 (R_670_ − R_550_))	(Rouse et al., 1974)
Simple ratio	SR	R800/R670	(Birth and McVey, 1968)
Modified simple ratio	MSR	(R750 − R445)/(R705 − R445)	(Sims and Gamon, 2002)
Photochemical reflection index	PRI	(R531 − R570)/(R531 + R570)	(Gamon et al., 1992)
Red edge position	REP	RRE = R670+R780/2 REP = 700 + 40X (RRE − R700)/(R740 − R700)	(Guyot and Baret, 1988)
Blue/green index	BIG2	R450/R550	(Zarco-Tejada et al., 2005)
Plant senescence reflectance index	PSRI	(R680-R500)/R750	(Gitelson et al., 2001)

#### 2.4.2 Ceptometer

A ceptometer (model AccuPAR LP-80, Decagon Devices, Pullman, USA) was utilized to measure the leaf area index (LAI) twice in early flowering and the middle of grain filling stages (LAI A and LAI B) within ±2 h of solar noon under cloudless conditions.

### 2.5 Salinity tolerance/sensitive indices

The grain yield means of 40 genotypes for non-saline soil (Yp) and saline soil (Ys) over two seasons were obtained to calculate the STIs. Based on these means, the stress tolerance indices were analyzed by the *i*PASTIC software (Pour-Aboughadareh et al., 2019). Additionally, Microsoft Excel calculates the newest index, CSI (Sabouri et al., 2022). Consequently, salinity tolerance/sensitive indices and equations are illustrated in **Table 3**. In addition, the grain yield means of non-saline soil (Yp) and saline soil (Ys) and grain yield over two sites over 2 years GY_E_ of 40 genotypes, and salinity tolerance/sensitive indices are shown in **Table 4**.

**Table 3:** Salinity tolerance/sensitive indices and equations.

Stress index	Formula	Desirable value	Reference
Tolerance	*TOL* = *Yp* − *Ys*	Minimum	(Rosielle and Hamblin, 1981)
Stress susceptibility index	$SSI=\frac{1-\left(Ys/Yp\right)}{1-\left(\bar{\text{Y}}\text{s}/\bar{\text{Y}}\text{p}\right)}$	Minimum	(Fischer and Maurer, 1978)
Geometric mean productivity	$GMP=\sqrt{Yp\times Ys}$	Maximum	(Fernandez, 1992)
Stress tolerance index	$STI=\frac{Ys\times Yp}{{\left(\bar{\text{Y}}\text{s}\right)}^{2}}\;$	Maximum	(Fernandez, 1992)
Harmonic mean	$HM=\frac{2\left(Ys\times Yp\right)}{Ys+Yp}$	Maximum	(Bidinger et al., 1987)
Mean productivity	$MP=\frac{Yp+Ys}{2}$	Maximum	(Rosielle and Hamblin, 1981)
Yield index	$YI=\frac{Ys}{\bar{\text{Y}}\text{s}}$	Maximum	(Gavuzzi et al., 1997)
Yield stability index	$YSI=\frac{Ys}{\text{Yp}}$	Maximum	(Bouslama and Schapaugh, 1984)
Relative stress index	$RSI=\frac{\left(Ys/Yp\right)}{\left(Ys/Yp\right)}$	Maximum	(Fischer and Wood, 1979)
Combination of significant indices	$CSI=\frac{1}{2}(\sum_{j}^{n}rYp.index_{j}\times index_{ij}\;+\sum_{j}^{n}rYs.index_{j}\times index_{ij})$	Maximum	(Sabouri et al., 2022)

**Table 4:** Grain yield means of non-saline sites Yp and saline soil sites Ys and grain yield over two sites for 2 years GY_E_ of 40 genotypes and salinity tolerance/sensitive indices.

Genotype	Yp	Ys	TOL	MP	GMP	HM	SSI	STI	YI	YSI	RSI	CSI	Rank	GY_E_
1	9,313	7,983	1,330	8,648	8,622	8,596	0.44	0.86	1.26	0.86	1.26	16,469.4	1	8,648^† a^
2	9,464	6,866	2,598	8,165	8,061	7,958	0.85	0.75	1.09	0.73	1.07	15,401.6	29	8,605 ^ab^
3	8,715	5,977	2,738	7,346	7,217	7,091	0.98	0.60	0.95	0.69	1.01	13,792	31	8,432 ^abc^
4	9,236	5,736	3,500	7,486	7,278	7,077	1.18	0.61	0.91	0.62	0.92	13,913.9	34	8,374 ^abc^
5	7,961	5,431	2,530	6,696	6,575	6,457	0.99	0.50	0.86	0.68	1.01	12,565.1	22	8,320 ^abc^
6	9,664	6,417	3,248	8,040	7,875	7,712	1.05	0.71	1.01	0.66	0.98	15,049.6	12	8,218 ^a-d^
7	7,995	6,092	1,903	7,044	6,979	6,915	0.74	0.56	0.96	0.76	1.12	13,333.1	21	8,188 ^a-e^
8	10,857	5,225	5,633	8,041	7,531	7,054	1.61	0.65	0.83	0.48	0.71	14,425.7	37	8,168 ^a-e^
9	9,319	6,257	3,062	7,788	7,636	7,487	1.02	0.67	0.99	0.67	0.99	14,592.2	2	8,165 ^a-e^
10	8,775	6,896	1,879	7,836	7,779	7,723	0.67	0.70	1.09	0.79	1.16	14,860.8	11	8,094 ^a-e^
11	10,152	6,036	4,116	8,094	7,828	7,571	1.26	0.71	0.95	0.59	0.88	14,967.9	33	8,094 ^a-e^
12	9,737	6,700	3,038	8,218	8,077	7,938	0.97	0.75	1.06	0.69	1.01	15,434.1	36	8,069 ^a-e^
13	8,222	5,909	2,313	7,065	6,970	6,876	0.88	0.56	0.93	0.72	1.06	13,317.7	8	8,041 ^a-e^
14	9,640	5,982	3,659	7,811	7,594	7,382	1.18	0.66	0.95	0.62	0.91	14,516.5	6	8,040 ^a-e^
15	8,633	6,210	2,423	7,421	7,322	7,224	0.87	0.62	0.98	0.72	1.06	13,989.7	30	8,032 ^a-e^
16	8,887	6,709	2,178	7,798	7,722	7,646	0.76	0.69	1.06	0.75	1.11	14,752.2	40	8,014 ^a-e^
17	9,580	6,205	3,375	7892	7,710	7,532	1.10	0.68	0.98	0.65	0.95	14,736	23	7,999 ^a-e^
18	7,945	6,755	1,190	7350	7,326	7,302	0.47	0.62	1.07	0.85	1.25	13,993.8	19	7,937 ^a-e^
19	9,456	6,418	3,038	7937	7,790	7,646	1.00	0.70	1.01	0.68	1.00	14,886.5	32	7,924 ^a-f^
20	9,576	6,047	3,529	7811	7,609	7,413	1.15	0.67	0.96	0.63	0.93	14,545.7	17	7,892 ^a-f^
21	9,692	6,685	3,007	8188	8,049	7,912	0.97	0.75	1.06	0.69	1.02	15,380.7	10	7,836 ^a-f^
22	9,354	7,286	2,068	8320	8,255	8,191	0.69	0.79	1.15	0.78	1.15	15,771.4	20	7,811 ^a-f^
23	9,924	6,075	3,850	7999	7,764	7,536	1.21	0.69	0.96	0.61	0.90	14,843.8	14	7,811 ^a-f^
24	9,487	5,263	4,225	7375	7,066	6,770	1.39	0.58	0.83	0.55	0.82	13,516.3	16	7,798 ^a-f^
25	8,911	5,832	3,080	7371	7,209	7,050	1.08	0.60	0.92	0.65	0.96	13,777.6	28	7,797 ^a-f^
26	8,849	6,441	2,408	7645	7,549	7,455	0.85	0.66	1.02	0.73	1.07	14,424.3	38	7,794 ^a-f^
27	9,149	5,979	3,171	7564	7,396	7,232	1.08	0.63	0.95	0.65	0.96	14,135.3	9	7,787 ^a-f^
28	10,050	5,544	4,506	7797	7,464	7,146	1.40	0.64	0.88	0.55	0.81	14,279.1	26	7,645 ^a-f^
29	9,902	7,308	2,594	8605	8,507	8,410	0.82	0.83	1.16	0.74	1.09	16,252.9	35	7,612 ^a-f^
30	9,861	6,203	3,659	8032	7,821	7,615	1.15	0.70	0.98	0.63	0.93	14,949.8	27	7,564 ^a-f^
31	10,666	6,197	4,469	8432	8,130	7,839	1.30	0.76	0.98	0.58	0.86	15,547.5	4	7,486 ^a-f^
32	9,890	5,957	3,933	7924	7,676	7,435	1.24	0.68	0.94	0.60	0.89	14,675.5	15	7,421 ^b-f^
33	9,495	6,692	2,803	8094	7,971	7,851	0.92	0.73	1.06	0.70	1.04	15,231.6	24	7,375 ^b-f^
34	9,787	6,960	2,827	8374	8,253	8,135	0.90	0.78	1.10	0.71	1.05	15,770.2	25	7,371 ^c-f^
35	9,346	5,879	3,467	7613	7,412	7,218	1.15	0.63	0.93	0.63	0.93	14,169.5	18	7,350 ^c-f^
36	10,045	6,093	3,952	8069	7,823	7,585	1.22	0.71	0.96	0.61	0.89	14,957.4	3	7,346 ^b-f^
37	8,947	7,389	1,559	8168	8,130	8,093	0.54	0.76	1.17	0.83	1.22	15,531.3	13	7,065 ^def^
38	9,844	5,745	4,099	7795	7,520	7,256	1.30	0.65	0.91	0.58	0.86	14,381	7	7,043 ^def^
39	8,066	5,820	2,247	6943	6,851	6,761	0.87	0.54	0.92	0.72	1.06	13,090.7	39	6,943 ^ef^
40	8,276	7,753	523	8014	8,010	8,005	0.20	0.74	1.23	0.94	1.38	15,299.2	5	6,696 ^f^
CV%														12.12

^†^Mean values within the same column for each trait with the same lowercase letter are not significantly different according to the least significant difference (LSD) at P ≤ 0.05. The rank of genotypes was based on GY_E_ combined analysis. TOL, tolerance index; MP, mean productivity stress; STI, tolerance index; GMP, geometric mean productivity; HM, harmonic mean; SSI, stress susceptibility index; YI, yield index; YSI, yield stability index; RSI, relative stress index; CSI, combination of significant indices.

### 2.6 Statistical analyses

Analysis of variance (ANOVA) data were collected for all characters separately in seasons 2019/20 and 2020/21 over two sites (saline and non-saline soil). Combined data of grain yield over two sites and two seasons (environments) and genotype by environment (GGE) biplots for grain yield over environments were accomplished according to Yan et al. (2000) and Yan et al. (2007). Statistical analysis was conducted through GenStat 19th edition (VSN International Ltd., Hemel Hempstead, UK). Additionally, a GYT biplot model was created based on grain yield and other agronomic and physiological traits (e.g., Fv/Fm) averaged over the saline and non-saline soil sites and season 2019/20 of the 40 genotypes. Hence, a genotype by trait table was generated, and then GYT combinations were computed by multiplying GY and all traits because high values are desirable except for DH and DM; they are divided by GY for the same reason (the multiplication operation is opposite of division) (Yan and Frégeau-Reid, 2018). At the same time, the second season GY and SRI (e.g., NDVI) utilized as traits along with Na^+^ and K^+^ were measured in the saline soil experiment only. However, salinity tolerance index values were used to depict genotype by salt tolerance index (GSTI) biplots (Yan and Frégeau-Reid, 2008); data were normalized before analyses as follows:


$$Y_{ij}=\frac{T_{ij}-{\bar{\text{T}}}_{j}}{S_{j}}$$

where Y_ij_ is the standardized genotype value *i* for yield–trait combination *j*, T_ij_ is the original value of genotype *i* for yield–trait combination *j*, $\bar{\text{T}}$ _j_ is the mean of genotype *i* for yield–trait combination *j*, and S_j_ is the standard deviation for yield–trait combination *j*, by GenStat 19th edition.

The traits normalized in Microsoft Excel to make a radar chart are as follows:


$${\text{X}}_{N}=\frac{\left({\text{X}}_{\text{O}}-{\text{X}}_{\text{MIN}}\right)\;}{\left({\text{X}}_{\text{MAX}}-{\text{X}}_{\text{MIN}}\right)}$$

where X_N_ is the normalized value, X_O_ is the original value, and X_MAX_ and X_MIN_ are the minimum and maximum values of the trait, respectively. This procedure obtains the traits as unitless in the case where they are compared. Origin (Pro), version 2021 (Origin Lab Corporation, Northampton, MA, USA) was utilized to illustrate radar charts.

## 3 Results

### 3.1 The estimated trait summary and mean performance

A summary of the studied traits measured in the 2019/2020 season is revealed in **Table S2**. The results revealed different minimum, maximum, and mean performance and genotype mean squares (MS Geno.) of non-saline and saline locations. There is significant difference between genotypes of all characters in non-saline and saline conditions, except for BY, CT, harvest index (HI), and SM for a non-saline soil site, which is in contrast with CT and SM in saline soil condition. Additionally, the CV of non-saline soil ranged from 2.01 for DM to 20.65% for SM. However, in the saline soil condition, CV ranged from 1.6 for DM to 25.89% for SM. Moreover, the rank of genotypes according to their mean performance for all studied characters was revealed.

**Table S3** summarizes the SRIs and GYs estimated across non-saline and saline conditions in the 2020/2021 season. The data are minimum, maximum, and means of all SRIs and GYs. Additionally, the mean square of genotypes and significant differences among them in all SRI and GY except in non-saline soil sites’ blue/green index (BIG2), modified simple ratio (MSR), SR, LAI A, and LAI B. However, in the saline soil site's, there are no significant differences among genotypes for SRI, such as modified chlorophyll absorption reflectance index (MCARI), LAI A, LAI B, and K/Na. In addition, high-ranked genotypes according to SRI mean performance were demonstrated.

The means of two non-saline soil sites (Yp) and saline soil sites (Ys) for the 40 genotypes were calculated over both studied seasons. Grain yield means ranged from 7,945 kg h^−1^ for genotype 18 to 10,857 kg h^−1^ for genotype 8 of non-saline soil sites. At the same time, the saline soil sites range from 5,225 kg h^−1^ of genotype 8 to 7,983 kg h^−1^ of genotype 1 (**Figure S1**). The results revealed that genotypes 1, 40, 37, 29, 33, 34, and 22 recorded the highest means and lowest fluctuations across seasons. In contrast, genotypes 8, 11, 23, 24, 28, and 31 had high fluctuations across environments over seasons (**Figure S1**).

### 3.2 The studied trait combined data analyses

**Table 5** shows the combined ANOVA for the agronomic and physiological studied traits in the 2019/2020 season under non-saline and saline soil sites. The effect due to sites significantly varied from site to site for all traits except for Fv/Fm. In addition, the genotype component has a significant variation for all studied characters except SM. In comparison, the effect of genotype by sites for BY, DH, DM, HI, SM, CCI, and CT was insignificant in contrast with other traits.

**Table 5:** Analysis of variance (mean square) of agronomic and physiological traits of 40 genotypes evaluated under non-saline and saline soil sites in the 2019/2020 season.

Source of variation	DF	BY	DH	DM	Fv/Fm	GY	HI
ENV	1	1.36E+08^**^	8,166^**^	16,335^**^	0.000006^NS^	1.58E+08^**^	1301^**^
REP:ENV	4	21,945,141	24.667	16.338	0.003626	2,155,530	219.86
BLK:REP:ENV	54	15,134,375	28.743	26.861	0.002444	1,298,744	68.53
GEN	39	9,105,748^*^	85.646^**^	51.2^**^	0.002373^*^	1,775,095^**^	51.82^*^
ENV:GEN	39	8,000,302^NS^	6.786^NS^	4.805^NS^	0.002529^**^	1,801,470^**^	27.51^NS^
Residual	102	5,465,161	5.193	7.388	0.001429	1,011,245	33.23
CV (%)		14.86	2.35	1.86	5.34	13.59	11.97
Source of variation	DF	NDVI	PH	SM	CCI	CT	
ENV	1	0.41917^**^	2,767^**^	415,751^**^	170.33^**^	4.692^*^	
REP:ENV	4	0.005033	55.42	16,460	115.43	23.718	
BLK:REP:ENV	54	0.007038	71.61	5,185	30.74	3.455	
GEN	39	0.010857^**^	168.53^**^	7,872^NS^	37.29^**^	2.044^*^	
ENV:GEN	39	0.005503^*^	23.13^**^	6,528^NS^	17.28^NS^	1.564^NS^	
Residual	102	0.003382	11.46	6,172	10.95	1.217	
CV (%)		10.84	3.44	24.26	10.42	4.36	

DF, degrees of freedom; ENV, environment (sites by season); GEN, genotype; REP, replication; BLK, block; BY, biological yield; DH, days to heading; DM, days to maturity; Fv/Fm, chlorophyll fluorescence; GY, grain yield; CT, canopy temperature; HI, harvest index; NDVI, normalized difference vegetation index; PH, plant height; SM^2^, number of spikes per square meter; CCI, chlorophyll content index; CV, coefficient of variation; MS Geno., mean square of genotypes; * and **, significance levels of P≤0.05 and P≤0.01; NS, no significant difference.

Based on the combined data of non-saline and saline soil sites (environments) presented in **Table 6**, the environment significantly varied for all traits, e.g., grain yield and spectral reflectance indices. Furthermore, there is significant variation among genotypes examined in the 2020/2021 season for all traits except photochemical reflection index (PRI), plant senescence reflectance index (PSRI), red edge position (REP), SR, and LAI A. However, the interaction between environment and genotype was insignificant for most traits except BIG2, CI, GY, MSR, and NDVI.

**Table 6:** Analysis of variance (mean square) of agronomic traits and spectral reflectance indices of the 40 genotypes evaluated in both non-saline and saline soil sites 2020/2021 season.

Source of variation	DF	BIG2	CI	GY	LCI	MCARI
ENV	1	0.076932^**^	0.012939^*^	1.14E+09^**^	0.125417^**^	0.000986^**^
REP:ENV	4	0.004917	0.0198	48,250,956	0.002748	3.14E−05
BLK:REP:ENV	54	0.000575	0.005866	4,077,516	0.000995	9.44E−05
GEN	39	0.000614^**^	0.005434^*^	1,669,177^**^	0.001398^**^	0.000112^*^
ENV:GEN	39	0.000615^**^	0.005677^**^	1,895,697^**^	0.000771^NS^	7.37E−05^NS^
Residual	102	0.000362	0.003238	786,465	0.000638	6.79E−05
CV (%)		2.95	−7.43	10.76	4.92	14.67
Source of variation	DF	MCARI 1	MSR	NDVI	PRI	PSRI
ENV	1	0.055588^**^	874.4954^**^	0.089744^**^	0.00352^**^	0.000823^**^
REP:ENV	4	0.005258	55.1711	0.003795	0.000621	0.000232
BLK:REP:ENV	54	0.002963	0.7201	0.000866	3.61E−05	7.17E−06
GEN	39	0.003111^**^	0.1974^**^	0.000969^**^	1.78E−05^NS^	1.79E−05^NS^
ENV:GEN	39	0.001688^NS^	0.2015^**^	0.000716^*^	1.78E−05^NS^	1.79E−05^NS^
Residual	102	0.001396	0.1022	0.000454	2.03E−05	1.32E−05
CV (%)		14.67	5.09	13.17	2.87	−31.8
Source of variation	DF	REP	SR	TVI	LAI1A	LAI B
ENV	1	369.3083^**^	30,984.33^**^	101.93^**^	753.3372^**^	164.309^**^
REP:ENV	4	23.2046	1,946.206	8.339	36.0375	43.632
BLK:REP:ENV	54	0.3795	19.943	4.5	0.9701	2.426
GEN	39	0.3205^NS^	0.2446^NS^	4.764^**^	0.5044^NS^	0.981^NS^
ENV:GEN	39	0.3161^NS^	0.2177^NS^	2.825^NS^	0.3914^NS^	1.087^NS^
Residual	102	0.2573	0.2769	2.193	0.3817	1.095
CV (%)		0.07	2.54	5.29	11.75	23.38

DF, degrees of freedom; ENV, environment (sites by season); GEN, genotype; REP, replication; BLK, block; GY, grain yield. * and **, significance levels of P≤0.05 and P≤0.01; NS, no significant difference.

The combined analysis of variance for grain yield traits over all sites and seasons (four environments) is demonstrated in **Table S4**. The data revealed significant differences among environments, genotypes, and environments by genotypes with significance levels (*P*≤0.01) along with CV 12.12%.

### 3.3 The comparison of the estimated traits and contrasting sites in both seasons

**Figure 1:**
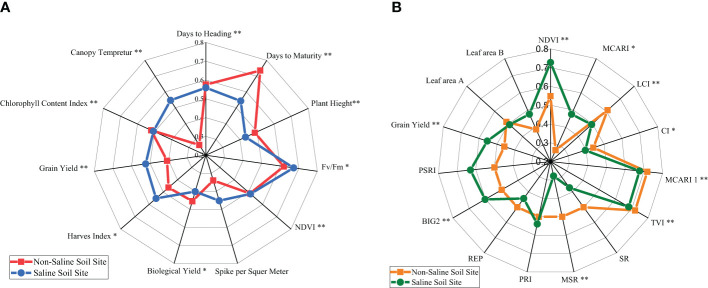
**(A)** Radar chart of the mean performance of the agronomic and physiological traits of 40 genotypes under non-saline and saline conditions evaluated in the 2019/2020 season. * and **, significance levels of *P*≤0.05 and *P*≤0.01, respectively, of genotype mean square. Fv/Fm, chlorophyll fluorescence; NDVI, normalized difference vegetation index. **(B)** Radar chart of the mean performance of grain yield (GY) and spectral reflectance indices along with LAI A and B, measured on 15 and 30 March 2021, for 40 genotypes under non-saline and saline conditions in the 2020/2021 season. * and **, significance levels of *P*≤0.05 and *P*≤0.01, respectively, of genotype mean square.

The mean performance of the agronomic and physiological traits of the 40 genotypes tested in non-saline soils versus saline soils in the 2019/2020 season is illustrated in a radar chart (**Figure 1A**). The results show that the same score was recorded for traits such as NDVI, days to heading (HD), and CCI for both sites, while the days to maturity, BY, and PH values of the non-saline soil site tend to be greater than those of the saline soil site. In contrast, GY, HI, CT, SM, and chlorophyll fluoresce (Fv/Fm) had the highest means for the saline site. The genotypes varied significantly (*P*≤ 0.01) in combined data for all revealed traits except SM.

The means of spectral reflectance indices and grain yield averaged over the 40 genotypes in the two sites (non-saline soils and saline soils) in the 2020/2021 season are displayed in **Figure 1B**. The saline soil site recorded higher values than the non-saline soil site for SRIs, such as NDVI, MCARI, PRI, BIG2, PSRI, leaf area B, and GY, while other indices are the opposite, except for triangular vegetation index (TVI), MCARI 1, and leaf area A, which had almost the same means as the two sites. The genotypes varied significantly (*P*≤ 0.01) in combined data of both sites for all shown traits and SRIs except PRI, REP, SR PSRI, and leaf areas A and B.

### 3.4 GGE biplots for grain yield over the four environments

**Figure 2:**
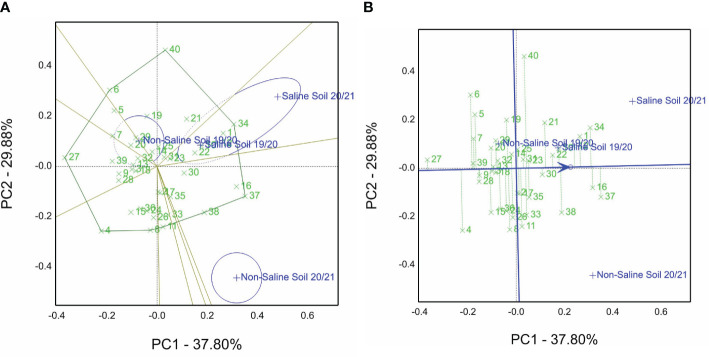
**(A)** Which-won-where GGE biplot view of the grain yield of 40 genotypes evaluated in non-saline and saline conditions in the 2019/2020 and 2020/2021 seasons (four environments). **(B)** Mean vs. stability view of the GGE biplot of 40 genotypes tested across non-saline and saline conditions in the 2019/2020 and 2020/2021 seasons (four environments).

**Figure 2A** presents the which-won-where of the GGE biplot view for the grain yield data of the 40 genotypes over all sites and seasons (four environments). Genotype 34 (1 close to 34) is the best one. It is located on the polygon vertices in sector content saline sites of two years. On the other hand, genotypes 40 and 6 are the winners in the non-saline soils in the first season of 2019/20, but genotype 37 (16 close to 37) is the winner in the non-saline soils’ second season of 2020/21. Principal components PC1 and PC2 explained 67.68% of the total variation of environments (E), genotypes (G), and G by E interaction. In the site of the saline soils (if we extend a vector from the biplot origin to points of saline sites), there was an acute angle between them. Thus, these sites are highly correlated in contrast to non-saline sites.

From the 40 genotype entries, genotype 37, followed by 16, 34, 1, and 12, is the top-ranked genotype evaluated under non-saline soils and saline soils of the cropping seasons, whereas genotype 27 ranked as the lowest genotype (**Figure 2B**). Furthermore, the five selected genotypes are located close to the line with an arrow and possess short projections. This means that the stable genotypes across the four investigated environments compared with genotypes 40, 4, and 6 pointed away from the average tester coordination (ATC) line.

### 3.5 The estimation of salt tolerance indices and their GT biplots

**Figure 3:**
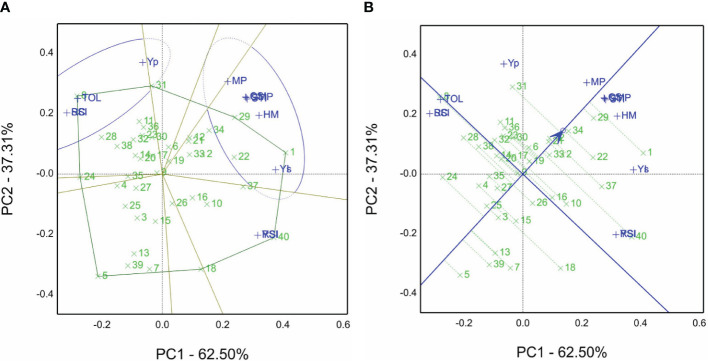
**(A)** Which-won-where view of the GSTI biplot of grain yield Yp in non-saline soil sites and Ys in saline soil sites in the 2019/2020 and 2020/2021 seasons of 40 genotypes with salinity tolerance indices, viz., tolerance index (TOL), mean productivity (MP), stress tolerance index (STI), geometric mean productivity (GMP), harmonic mean (HM), stress susceptibility index (SSI), yield index (YI), yield stability index (YSI), relative stress index (RSI), and combination of significant indices (CSI). **(B)** The average tester coordination view of the GSTI biplot of grain yield Yp in non-saline soil sites and Ys in saline soil sites in the 2019/2020 and 2020/2021 seasons of 40 genotypes with salinity tolerance indices, i.e., tolerance index (TOL), mean productivity (MP), stress tolerance index (STI), geometric mean productivity (GMP), harmonic mean (HM), stress susceptibility index (SSI), yield index (YI), yield stability index (YSI), relative stress index (RSI), and combination of significant indices (CSI).

The GT view was obtained to produce **Figure 3A** (GSTI) based on values of **Table 4** using the grain yield (Yp) of non-saline soil sites and saline soil sites (Ys) over both seasons, their combined data over four environments (GYE), and salt tolerance/susceptibility indices. The findings reveal that genotypes 1, 29, 31, 34, 22, and 12 recorded the highest means of grain yield (**Table 4**). Moreover, genotypes 1 and 29 are the winning genotypes for salinity indices such as Ys, YI, MP, STI, HM, CSI, and GMP. On the other hand, genotype 8 is the winning genotype based on RC, SSI, and TOL susceptibility indices and non-saline soil sites’ mean (Yp), and genotype 40 is the winner for YSI and RSI. Additionally, the sum of PC1 is 60.33% plus PC2 39.48%, equal to 99.8% of total variations, and it indicates the salinity tolerance indices STI and GY calculated from each other (**Figure 3A**).

According to the view of the GSTI biplot for average tester coordination, ATC is revealed in **Figure 3B**. The top-ranked genotypes are 1, followed by 29, 34, 22, and 31, while the poorest is genotype 5. Genotypes 29 and 34 had a strong performance and were close to the ATC line (short projection) compared with genotypes 40 and 18.

**Table S5** reveals that the genotypes’ ranks rely on STI and grain yield in non-saline soil (Yp) and saline soil sites (Ys). The results show that genotype 1 is the top-ranked one for Ys, MP, GMP, HM, STI, and YI. However, the opposing genotype 5 is the last ranked one for the same indices. On the other hand, genotype 8 recorded the lowest ranks for Ys, TOL, SSI, YI, YSI, and RSI. On the other hand, genotype 40 had the highest rank for the TOL, SSI (salinity tolerant), YSI, and RSI parameters. SR and AR are the sum and average of all ranks, and genotype 1 demonstrated the best one with values of 40 and 3.6. Nevertheless, genotype 24 recorded 391 and 35.5, respectively. The findings in **Figure 3A** confirmed these results.

The tester vector view of the GSTI biplot is depicted in **Figure S2**. The acute angle between STI vectors reflects the strength of the relationship or correlation and vice versa. For example, the angle between RSI and TOL indices indicates a negative correlation, while MP and GMP are highly positively correlated. These findings in **Figure S2** are confirmed by numerical values such as the correlation coefficient for RSI, and TOL is *r* = −0.98 in contrast to MP and GMP recorded *r* = 0.97. Moreover, the relation between RSI and YSI is identically confirmed by the same indices located on the same point (**Figure S2**).

### 3.6 The GYT biplots for agronomic and physiological traits and SRI of both seasons

**Figure 4:**
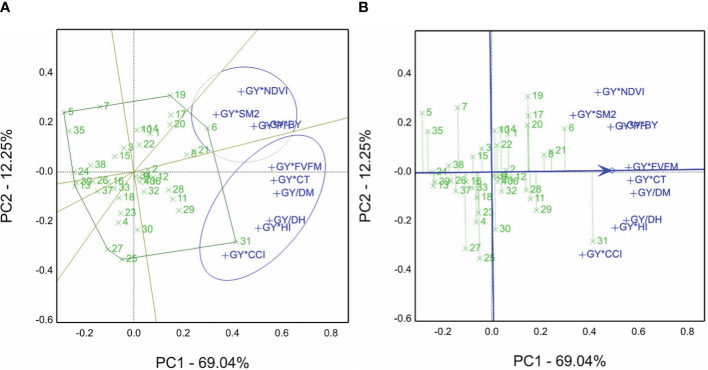
**(A)** Which-won-where view of the genotype by yield*trait (GYT) biplot of agronomic and physiological traits, e.g., BY, biological yield; DH, days to heading; DM, days to maturity; Fv/Fm, chlorophyll fluorescence; GY, grain yield; CT, canopy temperature; HI, harvest index; NDVI, normalized difference vegetation index; PH, plant height; SM2, number of spikes m^−1^; and CCI, chlorophyll content index, to create the combinations of 40 genotypes evaluated in normal and saline sites in the 2019/2020 season. **(B)** The average tester coordination view of the genotype by yield*trait (GYT) biplot of agronomic and physiological traits, e.g., BY, biological yield; DH, days to heading; DM, days to maturity; Fv/Fm, chlorophyll fluorescence; GY, grain yield; CT, canopy temperature; HI, harvest index; NDVI, normalized difference vegetation index; PH, plant height; SM2, number of spikes per square meter; and CCI, chlorophyll content index, to generate the combinations of 40 genotypes evaluated in normal and saline sites in the 2019/2020 season.

The GYT view is presented in **Figure 4A**. Based on the grain yield and other agronomic and physiological traits, the average of each genotype was evaluated under non-saline and saline sites in the 2019/2020 season. Hence, the GT table (two-way table) was generated, and then GYT combinations were normalized and calculated. Genotype 31 won the GY*Fv/Fm, GY*CT, GY/DH, GY/DM, GY*HI, and GY*CCI combinations. However, genotype 6 is the best for the GY*NDVI, GY*SM2, GY*PH, and GY*BY combinations. The sum of PC1 and PC2 accounted for 81.29% of total variations.

The GYT results are revealed in **Figure 4B**. The 40 genotypes’ ranking is 31>6>8>21>8>29, and genotype 5 is the lowliest genotype according to GYT combinations. However, the genotype placed close to the ATC line tended to be superior and had a balanced trait profile, e.g., genotypes 21 and 8 and vice versa, based on that view of the first season biplot data.

**Figure 5:**
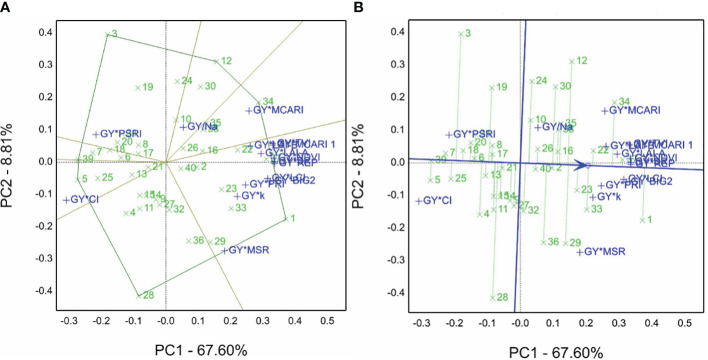
**(A)** Which-won-where view of the genotype by yield*trait (GYT) biplot of grain yield averaged in non-saline and saline soil sites with spectral reflectance indices to generate the combinations of 40 genotypes evaluated in the 2020/2021 season. **(B)** The average tester coordination view of the genotype by yield*trait (GYT) biplot of grain yield averaged non-saline and saline soil sites with spectral reflectance indices to generate the combinations of 40 genotypes evaluated in the 2020/2021 season.

For the 2020/2021 season, the grain yield averaged for the 40 genotypes and non-saline and saline sites were combined to produce the GYT combinations (data normalized before analyses) using SRIs. Genotype 1 was selected as the winner for most combinations, genotype 34 for the GY*MCARI combination, genotype 5 for the GY*CI combination, genotype 3 for the GY*PSRI combination, and genotype 12 for GY/Na combination (the minimum is the desirable value) (**Figure 5A**).

In the GYT biplot findings of the 2020/2021 season, the ATC (**Figure 5B**) demonstrated the best-ranked genotypes, e.g., genotype 1 followed by 37, 34, 22, 33. In contrast, genotype 5 had the lowest performance based on the GYT combinations with spectral reflectance indices, and genotypes 37 and 22 tended to be superior to 12 and 29. Thus, refer to the closeness and farness of the genotype location from the ATC line. The sum of contributions for PC1 and PC2 accounted for 76.14% of overall variations.

## 4 Discussion

Salinity tolerance varies from one specie to another, but the species’ tolerance mechanisms are similar to drought tolerance (Munns and Tester, 2008). Thus, we selected elite genotypes from CIMMYT drought trials, viz., SAWYT and SATYN, besides the yield potentiality trials, to identify salinity-tolerant genotypes evaluated under the open field conditions, as shown in **Table S1**. Several researchers (Sardouie-Nasab et al., 2014; Hinojosa et al., 2019; Mohammadi et al., 2022) have screened a large set of genotypes and then selected appropriate genotypes for field stress evaluation. They used different traits, agronomic characters, physiological traits, spectral reflectance indices, and STIs. In this study, relying on the GGE approach, genotypes 34 and 1 are the best for saline soil sites (**Figure 2A**). Genotypes 1 and 29 (**Figure 3A**) and genotype 34 (**Figure 3B**) are the best from the GSTI view. Genotype 1 is the best from the GYT view with SRI (**Figures 5A, B**). Therefore, genotype 1 could be identified as salt tolerant based on the STI and SRI results, shown in **Figures 3A, B**, **5A, B** and **Table S5**.

Salinity tolerance is a complex phenomenon controlled by several physiological functions and genetic factors (Gizaw et al., 2018) and influenced by growth stages and open field conditions (Haq et al., 2010; Oyiga et al., 2016; Tao et al., 2021). The appropriate design, alpha lattice, was used to reduce the experimental error generated and analyzed by GenStat, especially, in salinity-affected fields (acquired for genotype evaluation). Findings of grain yield data presented in **Table S4** (combined) over the four studied environments showed significant differences for environments, genotypes, and their interaction, which are similar to other reports (Ali et al., 2012; Enyew et al., 2021; Msundi et al., 2021; El-Hendawy et al., 2022). Additionally, similar findings were pointed out for the combined data and GGE biplot by (Enyew et al., 2021; Darwish et al., 2022; Darwish et al., 2023). **Tables 5**, **6** show significant differences in genotypes, environments, and their interactions for most studied traits over season by season separately. These findings agree with the results of agronomic traits (Enyew et al., 2021) and chlorophyll fluorescence (Fv/Fm) in quinoa crop (Hinojosa et al., 2019). In contrast, spectral reflectance index results agree with other reports (Prasad et al., 2007; El-Hendawy et al., 2019; Sun et al., 2019) regarding BIG2, CI, MSR, and NDVI as shown in **Table 6**.

The GGE, GT, and GYT models facilitate the mission of plant breeders to select tolerant genotypes for biotic and abiotic stresses. Based on the grain yield, other agronomic and physiological traits were averaged over the non-saline and saline soil sites, a genotype by trait table was generated, and then GYT combinations were normalized and calculated. Multiplication of GY and all traits was done compute the combinations because high values are desirable except for DH and DM. Multiplication of GY and all traits was done to compute the combinations because high values are desirable. However, DH and DM are divided by GY for the same reason (the multiplication operation is the opposite of division) (Yan and Frégeau-Reid, 2018). This study used GT analysis to address salinity tolerance indices generated from the *i*PASTIC online software (**Table 4**) and produced the GSTI biplots shown in **Figure 3A**, **Figure S2**, and **Figure 3B** and then identified genotypes 1 and 29 as salt-tolerant genotypes, while genotype 40 had yield stability. These findings agreed with the results of other reports (Yan and Frégeau-Reid, 2008; Mohammadi, 2019; Msundi et al., 2021; Santana et al., 2021; Zulfiqar et al., 2021). Salinity tolerance indices were calculated using the *i*PASTIC application based on grain yield in non-saline and saline soil sites over the years, and all the indices were employed to generate the GSTI biplots shown in **Figure 3A** and **Figure S2**. Other researchers, in this regard, obtained similar results (Mohammadi, 2019; Santana et al., 2021; Sabouri et al., 2022).

All traits measured of 40 genotypes were used to compare non-saline soil and saline soil sites. The traits data normalized by maximum and minimum values (to convert the raw data of traits into unitless values) and averaged of traits in a radar chart, e.g., for GY of the saline site recorded average higher than non-saline site in **Figures 1A, B** it may reflect the amount of variation in saline sites. These findings were similar to the results of several agronomic and physiological traits recorded by Al-Ashkar et al. (2019); Yang et al. (2020); Mohan et al. (2021); Yang et al. (2022), and Rebouh et al. (2023). In the same context, the wheat nitrogen deficit did not impact the Fv/Fm ratio (Gioia et al., 2015). However, sowing depth influences the grain yield of wheat (Amram et al., 2015).

The salt-tolerant genotype may have a minimal Na^+^ concentration, a higher K^+^ accumulation, a nicely maintained osmotic pressure in its roots and shoot, and maximal photosystem (PSII) activities, producing higher biomass specifically under salinity stress (Oyiga et al., 2016; Quamruzzaman et al., 2022). Accordingly, genotype 1 possesses a higher accumulation of K^+^ and the best rank of GY in saline soil sites in both seasons (**Tables S2**, **S3**). However, genotype 10 recorded the lowest Na^+^ concentration and K^+^:Na^+^ ratio and BY in the saline soil site (**Table S3**). These findings are consistent with those obtained in other reports (Oyiga et al., 2016; Morsy et al., 2022; Quamruzzaman et al., 2022). Genotype 12 was the best according to the GY/Na^+^ combination (**Figure 5A**), while genotype 1 was the best from the GY*K^+^ combination of the GYT biplot.

Remote sensing technologies and spectral instruments create valuable spectral information in many wavelength bands throughout the electromagnetic spectrum, particularly visible, near-infrared, and shortwave, and provide spectral reflectance indices. These approaches are becoming extremely powerful tools for identifying chemical and physical plant structures and functions by non-destructive methods and rapid and precise measurements (Reynolds et al., 2012; Bruning et al., 2020; El-Hendawy et al., 2022). Additionally, GGE, GT, and GYT biplots are other powerful tools in plant breeding for screening many genotypes and identify the best one, specifically under stress conditions. For example, researchers (Mohammadi et al., 2022) screened 220 durum wheat genotypes for drought tolerance. They used GT biplots to identify drought-tolerant genotypes; authors (Enyew et al., 2021) used GGE biplots to discriminate and select among 320 sorghum genotypes. At the same time, other researchers (Santana et al., 2021) used spectral reflectance indices and GT biplots to identify high-yielding corn genotypes evaluated under low- and high-nitrogen applications. Researchers (Elfanah et al., 2023) pointed out the selection of salt-tolerant wheat genotypes based on pots and lysimeter systems (sandy soil) identified employing STI and SRI parameters. In the current study, genotype 1 is the best one from the GYT view with SRI (**Figures 5A, B**) in saline soil (clay field).

Salinity stress reduces chlorophyll fluorescence (Fv/Fm), CCI, and NDVI (Oyiga et al., 2016; Quamruzzaman et al., 2022). However, the CCI value increased in plants under salinity conditions (Hasanuzzaman et al., 2018; Quamruzzaman et al., 2022). This value reflects the degradation of chlorophyll in salt-treated plants as well as reduced cell size and concentration of chlorophyll content in mesophyll tissues. In the present study, genotype 31 is the best one from the GYT biplot view based on GY*Fv/Fm, GY*CT, and GY*CCI, while it is genotype 6 in the same view for GY*NDVI (**Figures 4A, B**).

## 5 Conclusions

In conclusion, we used different selection criteria, physiological parameters, and spectral reflectance indices and estimated salinity tolerance indies simultaneously with grain yield. In this study, the results demonstrated significant differences (p≤0.01) among the environments, genotypes, and their interaction for GY evaluated in the four environments. Moreover, in the first season, the traits GY, PH, HI, CCI, chlorophyll fluorescence parameter Fv/Fm, and NDVI were measured in contrasting salinity environments. Additionally, significant differences were detected among environments, genotypes, and their interaction for grain yield along with SRIs, e.g., BIG2, curvature index (CI), NDVI, and MSR. Moreover, based on the GGE approach, genotypes 34 and 1 are the best performing in saline soil sites. Genotypes 1 and 29 and genotype 34 are the best from the GSTI biplot. Genotype 1 is the best from the GYT method with spectral reflectance indices. Therefore, we can identify genotype 1 as salt tolerant based on the results of GSTI and SRI and recommend including it in salinity breeding programs.

## Data availability statement

The raw data supporting the conclusions of this article will be made available by the authors, without undue reservation.

## Author contributions

All authors listed have made a substantial, direct, and intellectual contribution to the work, and approved it for publication.

## Supplementary material

Supplementary Figure 1 The tester vector view of GSTI biplot view of grain yield Yp in normal sites and Ys in stress sites in season 2019/2020 and 2020/2021 of 40 genotypes with salinity tolerance indices, e.g., Tolerance index TOL, Mean Productivity MP, Stress Tolerance Index STI, Geometric Mean Productivity GMP, Harmonic Mean HM, Stress Susceptibility Index SSI, Yield Index YI, Yield Stability Index YSI, Relative Stress Index RSI, and Combination of Significant Indices CSI.

Supplementary Figure 2 Correlation coefficient and diagram of grain yield Yp and Ys in non-saline and saline soil sites, respectively, averaged across 2019/2020 and 2020/2021 seasons with stress tolerance/sensitive indices STI.
