# Supplementary material for: Hyperspectral reflectance and agro-physiological traits for field identification of salt-tolerant wheat genotypes using the genotype by yield*trait biplot technique
Source: Front Plant Sci. 2023 Aug 2;14:1165113. doi: 10.3389/fpls.2023.1165113 (PMC10434226; doi:10.3389/fpls.2023.1165113)
Supplement: Supplementary file 1 [file DataSheet_1.zip › Supplemental materials/Supplimental Figures.docx]

**FIGURE 1S**

**FIGURE 1S** Bar plot for grain yield mean performance of forty genotypes across normal (Yp) and salinity (Ys) sites over both the 2019/20 and 2020/21 seasons, respectively.


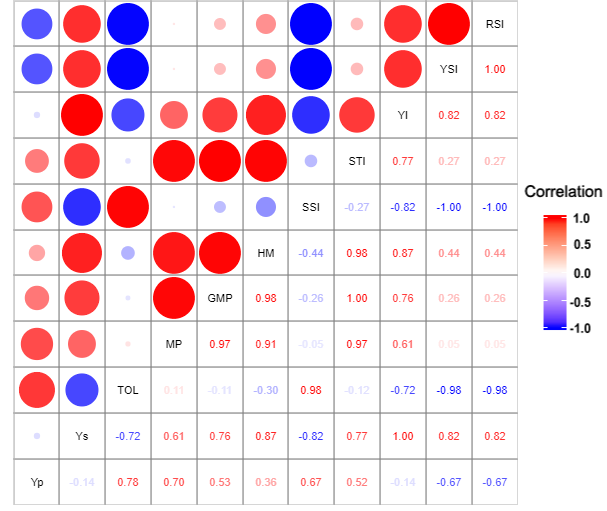
**FIGURE 2S**

**FIGURE 2S** Correlation coefficient and diagram of grain yield Yp and Ys normal and stress, respectively, averaged across 2019/20 and 2020/21 seasons with stress tolerance/sensitive indices STI.
