# Supplementary material for: Hyperspectral reflectance and agro-physiological traits for field identification of salt-tolerant wheat genotypes using the genotype by yield*trait biplot technique
Source: Front Plant Sci. 2023 Aug 2;14:1165113. doi: 10.3389/fpls.2023.1165113 (PMC10434226; doi:10.3389/fpls.2023.1165113)
Supplement: Supplementary file 1 [file DataSheet_1.zip › Supplemental materials/Supplimental Tables.docx]

**TABLE S1** Trial name, and pedigree along with selection history of selected forty genotypes to evaluation under normal and salinity conditions in seasons 2019/20 and 2020/21

| Ser | trial name 2018-19 | Pedigree | Selection history |
| --- | --- | --- | --- |
| 1 | 26 ^th^ SAWYT # 28 | BORL14*2/3/WBLL1*2/TUKURU//CROSBILL #1 | CMSS12B00631T-099TOPY-099M-0SY-4M-0WGY |
| 2 | 26 ^th^ SAWYT # 23 | WORRAKATTA/2*PASTOR/6/KAUZ/5/PAT10/ALD//PAT72300/3/PVN/4/BOW/7/BAJ #1/3/KIRITATI//ATTILA*2/PASTOR | CMSS12B00481S-099M-0SY-18M-0WGY |
| 3 | 26 ^th^ SAWYT # 40 | KACHU//WBLL1*2/BRAMBLING*2/6/ROLF07*2/5/REH/HARE//2*BCN/3/CROC_1/AE.SQUARROSA (213)//PGO/4/HUITES | CMSS12B00800T-099TOPY-099M-0SY-32M-0WGY |
| 4 | 26 ^th^ SAWYT # 35 | WBLL1*2/BRAMBLING/4/BABAX/LR42//BABAX*2/3/SHAMA*2/5/BECARD/QUAIU #1 | CMSS12B00714T-099TOPY-099M-0SY-18M-0WGY |
| 5 | 8 ^th^ SATYN # 2 | SOKOLL/WBLL1/5/W15.92/4/PASTOR//HXL7573/2*BAU/3/WBLL1 | PTSS11Y00209S-099B-099Y-099B-39Y-020Y-0B |
| 6 | 8 ^th^ SATYN # 12 | PBL94.14.30/4/PASTOR//HXL7573/2*BAU/3/WBLL1/5/BABAX/LR42//BABAX/3/ER2000 | PTSS12SHB00003T-0TOPB-099Y-099B-6Y-020Y-0B |
| 7 | 8 ^th^ SATYN # 11 | BAV92/SERI | CMSS96Y04084S-0Y-1B-93TLA-0B-0Y-106B-0Y-0Y-0Y-0Y |
| 8 | A – 4 ^th^ ESWST # 2 | SHORTENED SR26 TRANSLOCATION//2*WBLL1*2/KKTS/3/BECARD | CMSS08Y01115T-099M-099Y-099M-099NJ-14WGY-0B-0EG |
| 9 | A –4 ^th^ ESWST # 16 | SUP152/6/OASIS/5*BORL95/5/CNDO/R143//ENTE/MEXI75/3/AE.SQ/4/2*OCI | CMSA11Y00485S-099Y-099M-099NJ-099NJ-22WGY-0B-0EG |
| 10 | A –4 ^th^ ESWST # 24 | WHEAR/SOKOLL/3/TRCH/SRTU//KACHU | CMSS10Y00201S-099Y-099M-099NJ-099NJ-2WGY-0B-0EG |
| 11 | A – 4 ^th^ ESWST # 12 | BECARD/FRNCLN/3/KACHU #1/KIRITATI//KACHU | CMSS11B00426S-099M-099NJ-099NJ-3WGY-0B-0EG |
| 12 | A – 4 ^th^ ESWST # 1 | MUNAL*2/WESTONIA | CMSS08Y00833T-099TOPM-099Y-099M-099NJ-099NJ-14WGY-0B-0EG |
| 13 | A – 4 ^th^ ESWST # 5 | WHEAR//2*PRL/2*PASTOR/3/WAXBI/4/COPIO | CMSS11Y00722T-099TOPM-099Y-099M-099NJ-099NJ-6WGY-0B-0EG |
| 14 | A – 4 ^th^  ESWST # 26 | KACHU/SAUAL*2/8/ATTILA*2/PBW65/6/PVN//CAR422/ANA/5/BOW/CROW/BUC/PVN/3/YR/4/TRAP#1/7/ATTILA/2*PASTOR | CMSS10B01031T-099TOPY-099M-099NJ-099NJ-18WGY-0B-0EG |
| 15 | A – 4 ^th^ ESWST # 10 | SAUAL/YANAC//SAUAL*2/3/TACUPETO F2001/BRAMBLING*2//KACHU | CMSS11Y01150T-099TOPM-099Y-099M-0SY-11M-0WGY-0EG |
| 16 | D – ESWFT # 6 | BORLAUG100 F2014 | CMSS06Y00605T-099TOPM-099Y-099ZTM-099Y-099M-11WGY-0B-0MEX-0EG |
| 17 | D – ESWFT # 5 | BAJ #1 | CGSS01Y00134S-099Y-099M-099M-13Y-0B-0EG |
| 18 | D – ESWFT # 10 | BABAX/LR42//BABAX/3/ER2000*2/4/SRN/AE.SQUARROSA (358)//MILAN/SHA7 | CMSA10Y00112T-099B-050Y-099ZTM-099NJ-099NJ-5RGY-0B-0EG |
| 19 | 26 ^th^ HRWYT # 38 | ONIX/KBIRD*2//KFA/2*KACHU | CMSS12B00984T-099TOPY-099M-099NJ-099NJ-8RGY-0B |
| 20 | 26 ^th^ HRWYT # 39 | ONIX/KBIRD*2//KFA/2*KACHU | CMSS12B00984T-099TOPY-099M-099NJ-099NJ-15RGY-0B |
| 21 | 26 ^th^  HRWYT # 44 | WBLL1*2/BRAMBLING//WBLL1*2/SHAMA/3/WBLL1*2/BRAMBLING*2/4/KACHU/KIRITATI | CMSS12B00991T-099TOPY-099M-0SY-39M-0RGY |
| 22 | 26 ^th^ HRWYT # 40 | ONIX/KBIRD*2//KFA/2*KACHU | CMSS12B00984T-099TOPY-099M-099NJ-099NJ-18RGY-0B |
| 23 | 26 ^th^ HRWYT # 41 | ONIX/KBIRD*2//KFA/2*KACHU | CMSS12B00984T-099TOPY-099M-099NJ-099NJ-20RGY-0B |
| 24 | 26 ^th^ HRWYT # 23 | MUTUS*2/KINGBIRD #1/3/KSW/SAUAL//SAUAL/4/MUTUS//WBLL1*2/BRAMBLING/3/WBLL1*2/BRAMBLING | CMSS12Y01067T-099TOPM-099Y-099M-099NJ-099NJ-7RGY-0B |
| 25 | 26 ^th^ HRWYT # 15 | KSW/5/2*ALTAR 84/AE.SQUARROSA (221)//3*BORL95/3/URES/JUN//KAUZ/4/WBLL1/6/PRL/2*PASTOR*2//FH6-1-7/7/KIRITATI//PRL/2*PASTOR/5/OASIS/SKAUZ//4*BCN/3/PASTOR/4/KAUZ*2/YACO//KAUZ/6/KIRITATI//PRL/2*PASTOR | CMSS12Y00903T-099TOPM-099Y-099M-099NJ-099NJ-7RGY-0B |
| 26 | 26 ^th^ HRWYT # 13 | WBLL1*2/BRAMBLING/4/BABAX/LR42//BABAX*2/3/SHAMA*2/6/BABAX/LR42//BABAX*2/3/KUKUNA/4/CROSBILL #1/5/BECARD | CMSS12Y00849T-099TOPM-099Y-099M-099NJ-099NJ-8RGY-0B |
| 27 | 26 ^th^ HRWYT # 21 | K9644//KIRITATI/2*TRCH/3/BECARD/QUAIU #1/4/BABAX/LR42//BABAX/3/ER2000 | CMSS12Y01031T-099TOPM-099Y-099M-099NJ-099NJ-19RGY-0B |
| 28 | 26 ^th^ HRWYT # 18 | BLOUK #1/KINGBIRD #1*2//BECARD/QUAIU #1 | CMSS12Y00979T-099TOPM-099Y-099M-099NJ-099NJ-5RGY-0B |
| 29 | 39 ^th^ ESWYT # 18 | NADI//TRCH/HUIRIVIS #1/3/NADI | CMSS12Y00880T-099TOPM-099Y-099M-0SY-17M-0WGY |
| 30 | 39 ^th^ ESWYT # 6 | SUP152/QUAIU #2//BECARD/QUAIU #1 | CMSS11B00405S-099M-099NJ-099NJ-26WGY-0M |
| 31 | 39 ^th^ ESWYT # 22 | KIRITATI/WBLL1//2*BLOUK #1*2/3/KACHU #1/KIRITATI//KACHU | CMSS12Y00946T-099TOPM-099Y-099M-0SY-13M-0WGY |
| 32 | 39 ^th^ ESWYT # 34 | KACHU/BECARD//WBLL1*2/BRAMBLING/3/FRNCLN*2/TECUE #1 | CMSS12B00317S-099M-0SY-1M-0WGY |
| 33 | 39 ^th^ ESWYT # 10 | SUP152/AKURI//SUP152/3/MUCUY | CMSS12Y00300S-099Y-099M-0SY-4M-0WGY |
| 34 | 39 ^th^ ESWYT # 41 | CNO79//PF70354/MUS/3/PASTOR/4/BAV92*2/5/HAR311/6/BECARD/QUAIU #1/7/BECARD/QUAIU #1 | CMSS12B00640T-099TOPY-099M-0SY-14M-0WGY |
| 35 | 39 ^th^ ESWYT # 45 | KACHU//WBLL1*2/BRAMBLING*2/3/KACHU/KIRITATI | CMSS12B00801T-099TOPY-099M-0SY-36M-0WGY |
| 36 | 39 ^th^ ESWYT # 50 | FRET2*2/BRAMBLING//BECARD/3/WBLL1*2/BRAMBLING*2/4/BECARD/QUAIU #1 | CMSS12B00944T-099TOPY-099M-0SY-33M-0WGY |
| 37 | 39 ^th^ ESWYT # 36 | CIRO16/2*BORL14 | CMSS12B00569T-099TOPY-099M-0SY-53M-0WGY |
| 38 | 39 ^th^ ESWYT # 46 | KACHU//WBLL1*2/BRAMBLING*2/3/KACHU/KIRITATI | CMSS12B00801T-099TOPY-099M-0SY-46M-0WGY |
| 39 | MISR 3 (cultivar) | ATTILA*2/PBW65*2/KACHU | CMSS06Y00582T-099TOPM-099Y-099ZTM-099Y-099M-10WGY-0B-0EGY |
| 40 | Sakha 95 (cultivar) | PASTOR//SITE/ MO/3/CHEN/AEGILOPS SQUARROSA (TAUS)//BCN/4/WBLL1 | CMA01Y00158S-040POY-040M-030ZTM-040SY-26M-0Y-0SY-0S |

SAWYT, Semi-Arid Wheat Yield Trial; SATYN, Stress Adaptive Trait Yield N; A-ESWST, Elite Spring Bread Wheat Trial under Water stress Conditions; D-ESWFT, Elite Spring Bread Wheat Trial Favorable Conditions; HRWYT, High Rainfall Wheat Screening Trial; and ESWYT, Elite Spring Wheat Yield Trial

**TABLE S2** Summary of studied characters analyses and genotypes ranking for normal and saline soil sites in season 2019/20

| trait | BY | | DH | | DM | | Fv/Fm | | GY | | CT |
| --- | --- | --- | --- | --- | --- | --- | --- | --- | --- | --- | --- |
| site | NORMAL | STRESS | NORMAL | STRESS | NORMAL | STRESS | NORMAL | STRESS | NORMAL | STRESS | NORMAL |
| Min. | 13942 | 9835 | 92 | 83 | 144 | 129 | 0.59 | 0.63 | 6296 | 4989 | 22.83 |
| Max. | 21900 | 16585 | 110 | 99 | 160 | 145 | 0.76 | 0.75 | 10708 | 7623 | 29.05 |
| Mean | 18106 | 13313 | 103 | 91 | 154 | 138 | 0.71 | 0.71 | 8211 | 6580 | 25.50 |
| MS geno | NS | ** | ** | ** | ** | ** | * | * | * | ** | NS |
| CV | 15.29 | 13.53 | 2.35 | 2.34 | 2.01 | 1.64 | 6.02 | 4.56 | 14.61 | 11.58 | 4.91 |
| Genotype Rank | | | | | | | | | | | |
| 1 | 6 | 10 | 25 | 11 | 25 | 25 | 9 | 2 | 31 | 1 | 17 |
| 2 | 21 | 17 | 4 | 27 | 4 | 4 | 15 | 16 | 6 | 18 | 23 |
| 3 | 36 | 14 | 27 | 4 | 27 | 24 | 10 | 37 | 8 | 10 | 28 |
| 4 | 27 | 19 | 28 | 25 | 11 | 27 | 30 | 14 | 21 | 15 | 37 |
| 5 | 31 | 9 | 31 | 16 | 24 | 2 | 40 | 3 | 29 | 31 | 4 |
| 6 | 24 | 12 | 36 | 31 | 40 | 11 | 3 | 1 | 19 | 17 | 16 |
| 7 | 19 | 7 | 16 | 23 | 28 | 8 | 32 | 13 | 28 | 40 | 8 |
| 8 | 14 | 2 | 23 | 28 | 12 | 29 | 7 | 39 | 32 | 37 | 24 |
| 9 | 20 | 1 | 13 | 13 | 37 | 22 | 8 | 4 | 14 | 16 | 21 |
| 10 | 32 | 22 | 18 | 2 | 2 | 18 | 18 | 12 | 20 | 9 | 9 |
| trait | HI | | NDVI | | PH | | SM | | CCI | | CT |
| site | NORMAL | STRESS | NORMAL | STRESS | NORMAL | STRESS | NORMAL | STRESS | NORMAL | STRESS | STRESS |
| min | 38 | 40 | 0.44 | 0.26 | 84 | 82 | 216 | 146 | 23.42 | 16.94 | 25.16 |
| max | 54 | 61 | 0.65 | 0.65 | 118 | 104 | 356 | 503 | 37.89 | 40.28 | 26.16 |
| mean | 46 | 51 | 0.58 | 0.49 | 102 | 95 | 282 | 366 | 30.73 | 32.79 | 27.16 |
| MS geno | NS | * | ** | ** | ** | ** | NS | NS | ** | * | NS |
| CV | 12.44 | 11.55 | 7.54 | 14.1 | 3.53 | 3.32 | 20.65 | 25.89 | 8.75 | 11.68 | 3.7 |
| Genotype Rank | | | | | | | | | | | |
| 1 | 25 | 25 | 7 | 10 | 7 | 7 | 3 | 20 | 27 | 30 | 1 |
| 2 | 11 | 31 | 19 | 19 | 5 | 5 | 12 | 8 | 13 | 12 | 25 |
| 3 | 31 | 11 | 10 | 9 | 6 | 16 | 8 | 28 | 24 | 36 | 24 |
| 4 | 8 | 30 | 5 | 23 | 17 | 12 | 25 | 7 | 26 | 31 | 39 |
| 5 | 4 | 3 | 20 | 20 | 1 | 13 | 28 | 2 | 23 | 34 | 30 |
| 6 | 29 | 36 | 6 | 21 | 19 | 6 | 34 | 35 | 39 | 9 | 27 |
| 7 | 38 | 18 | 1 | 35 | 13 | 17 | 19 | 15 | 18 | 23 | 31 |
| 8 | 23 | 26 | 15 | 17 | 29 | 40 | 15 | 36 | 30 | 28 | 28 |
| 9 | 30 | 40 | 17 | 6 | 14 | 10 | 16 | 11 | 16 | 27 | 8 |
| 10 | 28 | 39 | 29 | 2 | 22 | 34 | 39 | 17 | 9 | 4 | 26 |

BY, biological yield; DH, days to heading; DM, days to maturity; Fv/Fm, Chlorophyll fluorescence; GY, grain yield; CT, Canopy temperature; HI, harvest index; NDVI, Normalized difference vegetation index; PH, plant height; SM, number of spikes m ^-1^; and CCI, chlorophyll content index; CV, coefficient of variation; MS Geno., Mean square of genotypes; *and ** Significant level of P≤0.05 and P≤0.01 and NS no significant difference.

**TABLE**  **S3** Summary of grain yield and spectral reflectance indices analyses and genotypes ranking for normal and saline soil sites in season 2020/21

| trait | BIG2 | | CI | | GY | | LCI | | MCARI | | MCARI1 | | MSR | |
| --- | --- | --- | --- | --- | --- | --- | --- | --- | --- | --- | --- | --- | --- | --- |
| site | NORMAL | STRESS | NORMAL | STRESS | NORMAL | STRESS | NORMAL | STRESS | NORMAL | STRESS | NORMAL | STRESS | NORMAL | STRESS |
| min | 0.66 | 0.55 | -0.85 | -0.90 | 8603 | 4309 | 0.472 | 0.444 | 0.044 | 0.043 | 0.695 | 0.576 | 0.445 | 3.40 |
| max | 0.66 | 0.67 | -0.68 | -0.69 | 11635 | 8218 | 0.577 | 0.532 | 0.064 | 0.073 | 0.784 | 0.778 | 0.574 | 4.95 |
| mean | 0.66 | 0.63 | -0.76 | -0.77 | 10422 | 6067 | 0.536 | 0.490 | 0.054 | 0.058 | 0.750 | 0.719 | 0.519 | 4.34 |
| MS geno | NS | * | * | * | ** | ** | * | * | * | NS | * | * | NS | ** |
| CV | 0.06 | 4.3 | -5.55 | -8.87 | 8.24 | 15.07 | 4.74 | 5.13 | 11.37 | 17 | 3.97 | 6.07 | 9.03 | 10.37 |
| Genotype Rank | | | | | | | | | | | | | | |
| 1 | 37 | 11 | 29 | 28 | 38 | 1 | 13 | 6 | 28 | 38 | 13 | 38 | 26 | 28 |
| 2 | 4 | 4 | 11 | 9 | 8 | 40 | 23 | 9 | 3 | 12 | 15 | 12 | 9 | 36 |
| 3 | 12 | 32 | 27 | 18 | 33 | 29 | 27 | 36 | 7 | 30 | 34 | 34 | 31 | 32 |
| 4 | 1 | 14 | 23 | 11 | 37 | 37 | 15 | 11 | 31 | 19 | 30 | 6 | 17 | 4 |
| 5 | 21 | 23 | 40 | 32 | 11 | 22 | 1 | 32 | 19 | 26 | 37 | 19 | 29 | 14 |
| 6 | 13 | 5 | 4 | 27 | 16 | 34 | 30 | 27 | 9 | 34 | 12 | 30 | 2 | 29 |
| 7 | 23 | 29 | 38 | 4 | 34 | 23 | 8 | 29 | 37 | 24 | 22 | 29 | 16 | 9 |
| 8 | 11 | 7 | 8 | 21 | 35 | 27 | 34 | 15 | 24 | 6 | 19 | 26 | 32 | 5 |
| 9 | 7 | 31 | 2 | 25 | 36 | 2 | 32 | 4 | 14 | 35 | 10 | 1 | 19 | 27 |
| 10 | 39 | 40 | 39 | 39 | 24 | 12 | 33 | 1 | 5 | 3 | 24 | 17 | 3 | 15 |
| trait | NDVI | | SR | | TVI |  | LAI A | | LAI B | | Na | K | K/Na |  |
| site | NORMAL | STRESS | NORMAL | STRESS | NORMAL | STRESS | NORMAL | STRESS | NORMAL | STRESS | STRESS | STRESS | STRESS |  |
| Min. | 0.72 | 0.67 | 31.58 | 8.50 | 26.49 | 21.64 | 6.25 | 1.61 | 3.34 | 0.77 | 0.72 | 2.60 | 1.52 |  |
| Max. | 0.79 | 0.76 | 32.41 | 10.33 | 29.91 | 29.61 | 8.21 | 3.83 | 7.44 | 5.10 | 3.28 | 6.37 | 4.21 |  |
| mean | 0.76 | 0.72 | 32.06 | 9.34 | 28.63 | 27.33 | 7.43 | 3.09 | 5.49 | 3.46 | 1.89 | 4.95 | 2.92 |  |
| MS geno. | * | ** | NS | NS | * | * | NS | NS | NS | NS | ** | * | NS |  |
| CV | 2.73 | 3.01 | 0.9 | 7.35 | 4 | 6.41 | 8.45 | 19.67 | 20 | 28.67 | 20.31 | 12.9 | 21.99 |  |
| Genotype Rank | | | | | | | | | | | | | |  |
| 1 | 27 | 6 | 37 | 6 | 13 | 38 | 20 | 27 | 24 | 27 | 10 | 1 | 10 |  |
| 2 | 23 | 3 | 4 | 15 | 15 | 34 | 19 | 1 | 1 | 1 | 3 | 15 | 23 |  |
| 3 | 32 | 1 | 12 | 3 | 37 | 12 | 14 | 10 | 23 | 22 | 2 | 25 | 32 |  |
| 4 | 19 | 17 | 1 | 17 | 34 | 6 | 24 | 21 | 22 | 40 | 4 | 28 | 13 |  |
| 5 | 30 | 9 | 21 | 8 | 22 | 19 | 9 | 34 | 11 | 12 | 23 | 29 | 40 |  |
| 6 | 34 | 34 | 13 | 1 | 19 | 30 | 25 | 32 | 3 | 34 | 16 | 37 | 33 |  |
| 7 | 9 | 32 | 23 | 12 | 30 | 29 | 38 | 22 | 34 | 30 | 18 | 7 | 16 |  |
| 8 | 15 | 12 | 11 | 18 | 10 | 17 | 23 | 29 | 9 | 32 | 40 | 13 | 27 |  |
| 9 | 1 | 36 | 7 | 21 | 12 | 1 | 22 | 33 | 14 | 7 | 32 | 23 | 7 |  |
| 10 | 8 | 18 | 39 | 37 | 32 | 26 | 28 | 9 | 20 | 24 | 9 | 36 | 9 |  |

NDVI, Normalized difference vegetation index; CV, coefficient of variation; MS Geno., Mean square of genotypes; *and ** Significant levels of P≤0.05 and P≤0.01, and NS no significant difference; LAI A and B, leaf area index; GY, grain yield

**TABLE S4** Combined analysis for grain yield over normal and salinity sites and seasons 2019/20 and 2020/21

| Source of variation | DF | SS | MS |
| --- | --- | --- | --- |
| ENV | 3 | 1381197844 | 460399281** |
| REP:ENV | 8 | 201625946 | 25203243 |
| BLK:REP:ENV | 108 | 290318055 | 2688130 |
| GEN | 39 | 71134097 | 1823951** |
| ENV:GEN | 117 | 207382037 | 1772496** |
| Residual | 204 | 183366420 | 898855 |
| CV | 12.12% |  |  |

DF, Degree of freedom; SS, Sum of squares; MS, mean square; ENV, Environment; GEN, Genotype; REP, replication; BLK, Block; CV, coefficient of variation; *and ** Significant level of P≤0.05 and P≤0.01 and NS no significant difference, respectively.

**TABLE S5** Genotypes rank based on grain yield for evaluated forty genotypes under normal and stress (Yp and Ys) sites and salinity tolerance/sensitive indices over seasons 2019/20 and 2020/21

| Genotype | Yp | Ys | TOL | MP | GMP | HM | SSI | STI | YI | YSI | RSI | SR | AR | SD |
| --- | --- | --- | --- | --- | --- | --- | --- | --- | --- | --- | --- | --- | --- | --- |
| 1 | 25 | 1 | 3 | 1 | 1 | 1 | 2 | 1 | 1 | 2 | 2 | 40 | 3.6 | 7.1 |
| 2 | 20 | 8 | 15 | 9 | 8 | 7 | 11 | 8 | 8 | 11 | 11 | 116 | 10.5 | 3.9 |
| 3 | 33 | 29 | 16 | 36 | 34 | 32 | 19 | 34 | 29 | 19 | 19 | 300 | 27.3 | 7.5 |
| 4 | 26 | 36 | 28 | 31 | 33 | 33 | 30 | 33 | 36 | 30 | 30 | 346 | 31.5 | 3.1 |
| 5 | 39 | 38 | 13 | 40 | 40 | 40 | 20 | 40 | 38 | 20 | 20 | 348 | 31.6 | 10.8 |
| 6 | 14 | 16 | 25 | 14 | 12 | 13 | 23 | 12 | 16 | 23 | 23 | 191 | 17.4 | 5.1 |
| 7 | 38 | 23 | 6 | 38 | 37 | 36 | 7 | 37 | 23 | 7 | 7 | 259 | 23.5 | 14.4 |
| 8 | 1 | 40 | 40 | 13 | 26 | 34 | 40 | 26 | 40 | 40 | 40 | 340 | 30.9 | 13.3 |
| 9 | 24 | 17 | 22 | 27 | 22 | 21 | 22 | 22 | 17 | 22 | 22 | 238 | 21.6 | 2.8 |
| 10 | 32 | 7 | 5 | 21 | 17 | 12 | 5 | 17 | 7 | 5 | 5 | 133 | 12.1 | 8.8 |
| 11 | 3 | 26 | 36 | 10 | 13 | 18 | 35 | 13 | 26 | 35 | 35 | 250 | 22.7 | 11.9 |
| 12 | 12 | 11 | 20 | 6 | 7 | 8 | 18 | 7 | 11 | 18 | 18 | 136 | 12.4 | 5.2 |
| 13 | 36 | 31 | 10 | 37 | 38 | 37 | 14 | 38 | 31 | 14 | 14 | 300 | 27.3 | 11.6 |
| 14 | 15 | 27 | 30 | 23 | 24 | 25 | 31 | 24 | 27 | 31 | 31 | 288 | 26.2 | 4.8 |
| 15 | 34 | 18 | 12 | 32 | 32 | 29 | 13 | 32 | 18 | 13 | 13 | 246 | 22.4 | 9.3 |
| 16 | 30 | 10 | 8 | 24 | 19 | 14 | 8 | 19 | 10 | 8 | 8 | 158 | 14.4 | 7.6 |
| 17 | 16 | 19 | 26 | 20 | 20 | 20 | 26 | 20 | 19 | 26 | 26 | 238 | 21.6 | 3.6 |
| 18 | 40 | 9 | 2 | 35 | 31 | 26 | 3 | 31 | 9 | 3 | 3 | 192 | 17.5 | 15.0 |
| 19 | 21 | 15 | 21 | 18 | 16 | 15 | 21 | 16 | 15 | 21 | 21 | 200 | 18.2 | 2.8 |
| 20 | 17 | 25 | 29 | 22 | 23 | 24 | 27 | 23 | 25 | 27 | 27 | 269 | 24.5 | 3.3 |
| 21 | 13 | 13 | 19 | 7 | 9 | 9 | 17 | 9 | 13 | 17 | 17 | 143 | 13.0 | 4.1 |
| 22 | 22 | 5 | 7 | 5 | 3 | 3 | 6 | 3 | 5 | 6 | 6 | 71 | 6.5 | 5.3 |
| 23 | 6 | 24 | 32 | 17 | 18 | 19 | 32 | 18 | 24 | 32 | 32 | 254 | 23.1 | 8.5 |
| 24 | 19 | 39 | 37 | 33 | 36 | 38 | 38 | 36 | 39 | 38 | 38 | 391 | 35.5 | 5.8 |
| 25 | 29 | 33 | 23 | 34 | 35 | 35 | 24 | 35 | 33 | 24 | 24 | 329 | 29.9 | 5.2 |
| 26 | 31 | 14 | 11 | 28 | 25 | 22 | 10 | 25 | 14 | 10 | 10 | 200 | 18.2 | 8.1 |
| 27 | 27 | 28 | 24 | 30 | 30 | 28 | 25 | 30 | 28 | 25 | 25 | 300 | 27.3 | 2.2 |
| 28 | 4 | 37 | 39 | 25 | 28 | 31 | 39 | 28 | 37 | 39 | 39 | 346 | 31.5 | 10.5 |
| 29 | 7 | 4 | 14 | 2 | 2 | 2 | 9 | 2 | 4 | 9 | 9 | 64 | 5.8 | 4.0 |
| 30 | 9 | 20 | 30 | 15 | 15 | 16 | 29 | 15 | 20 | 29 | 29 | 227 | 20.6 | 7.4 |
| 31 | 2 | 21 | 38 | 3 | 6 | 11 | 37 | 6 | 21 | 37 | 37 | 219 | 19.9 | 15.1 |
| 32 | 8 | 30 | 33 | 19 | 21 | 23 | 34 | 21 | 30 | 34 | 34 | 287 | 26.1 | 8.4 |
| 33 | 18 | 12 | 17 | 11 | 11 | 10 | 16 | 11 | 12 | 16 | 16 | 150 | 13.6 | 2.9 |
| 34 | 11 | 6 | 18 | 4 | 4 | 4 | 15 | 4 | 6 | 15 | 15 | 102 | 9.3 | 5.6 |
| 35 | 23 | 32 | 27 | 29 | 29 | 30 | 28 | 29 | 32 | 28 | 28 | 315 | 28.6 | 2.5 |
| 36 | 5 | 22 | 34 | 12 | 14 | 17 | 33 | 14 | 22 | 33 | 33 | 239 | 21.7 | 10.2 |
| 37 | 28 | 3 | 4 | 8 | 5 | 5 | 4 | 5 | 3 | 4 | 4 | 73 | 6.6 | 7.2 |
| 38 | 10 | 35 | 35 | 26 | 27 | 27 | 36 | 27 | 35 | 36 | 36 | 330 | 30.0 | 7.9 |
| 39 | 37 | 34 | 9 | 39 | 39 | 39 | 12 | 39 | 34 | 12 | 12 | 306 | 27.8 | 13.3 |
| 40 | 35 | 2 | 1 | 16 | 10 | 6 | 1 | 10 | 2 | 1 | 1 | 85 | 7.7 | 10.4 |

TOL, Tolerance index; MP, Mean Productivity Stress; STI, Tolerance Index GMP, Geometric Mean Productivity; HM, Harmonic Mean; SSI, Stress Susceptibility Index; YI, Yield Index; YSI, Yield Stability Index; and RSI, Relative Stress Index, SR, Sum of ranks; AR, Average of ranks; SD, Standard division of ranks
